# Supplementary material for: miR-210 Expression Is Strongly Hypoxia-Induced in Anaplastic Thyroid Cancer Cell Lines and Is Associated with Extracellular Vesicles and Argonaute-2
Source: Int J Mol Sci. 2023 Feb 24;24(5):4507. doi: 10.3390/ijms24054507 (PMC10002857; doi:10.3390/ijms24054507)
Supplement: Supplementary file 1 [file ijms-24-04507-s001.zip › ijms-2132397-supplementary.pdf]

Figure S1

A.

hsa-miR-210-3p

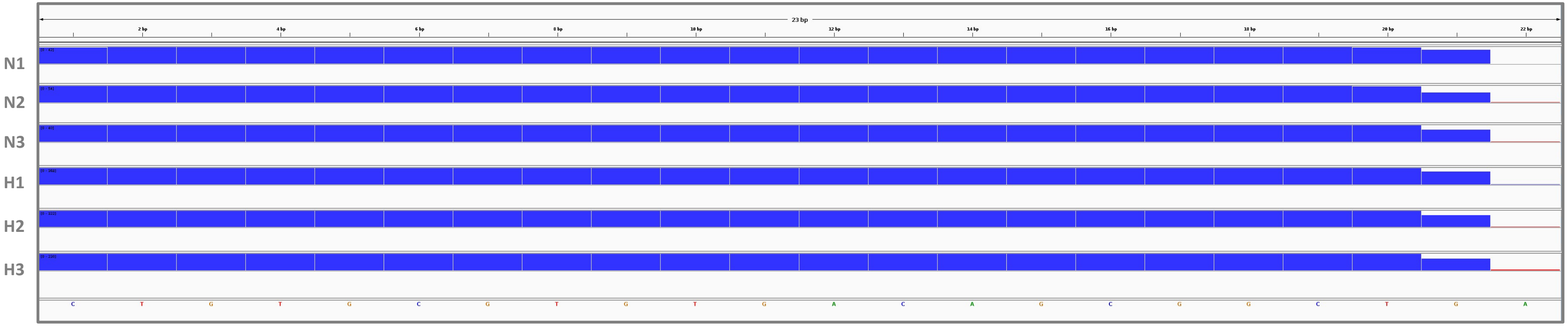

B.

hsa-premiR-210

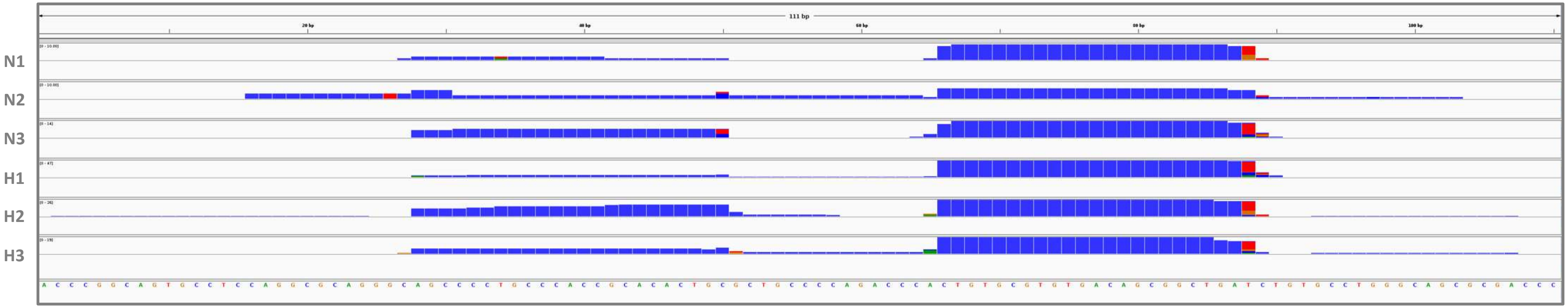

Figure S2

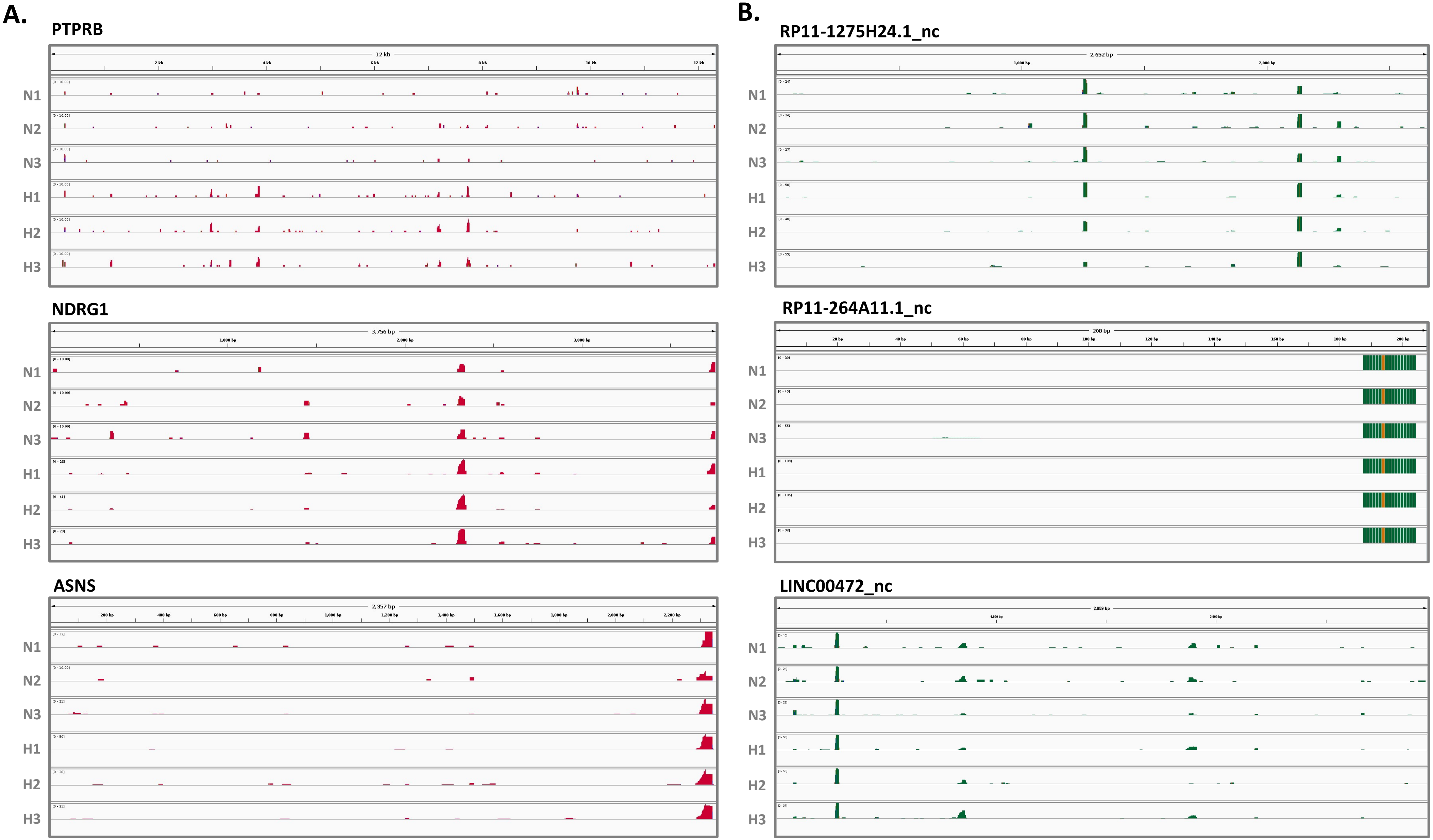
A.
PTPRB
12 kb
N1
N2
N3
H1
H2
H3
NDRG1
3,756 bp
N1
N2
N3
H1
H2
H3
ASNS
2,357 bp
N1
N2
N3
H1
H2
H3
B.
RP11-1275H24.1_nc
2,652 bp
N1
N2
N3
H1
H2
H3
RP11-264A11.1_nc
200 bp
N1
N2
N3
H1
H2
H3
LINC00472_nc
N1
N2
N3
H1
H2
H3

Figure S3

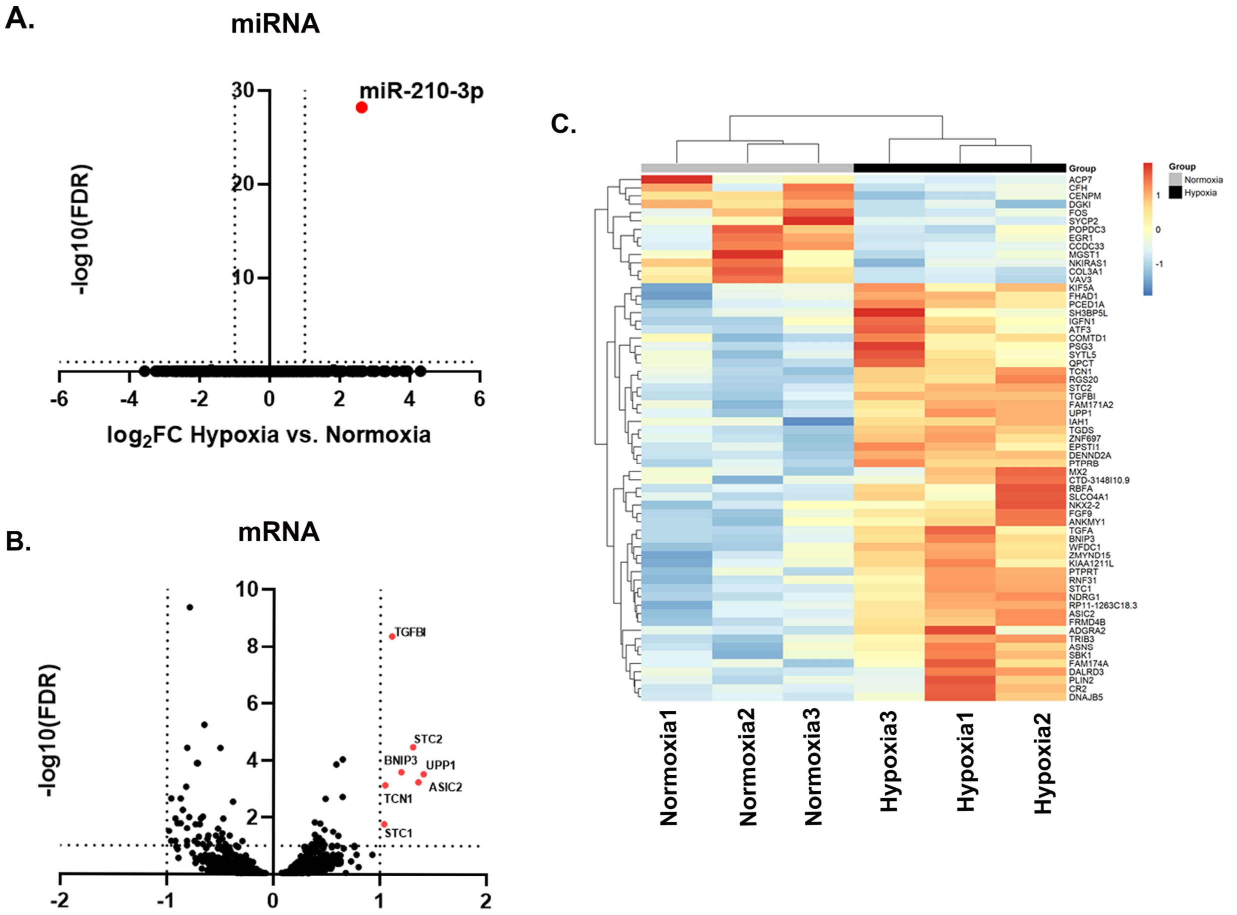
A.
miRNA
miR-210-3p
-log10(FDR)
30
20
10
-6
-4
-2
0
2
4
6
log$_2$FC Hypoxia vs. Normoxia
B.
mRNA
-log10(FDR)
10
8
6
4
2
-2
-1
0
1
2
TGFBI
STC2
BNIP3
UPP1
ASIC2
TCN1
STC1
C.
Group
Normoxia
Hypoxia
1
0
-1
ACP7
CFH
CENPM
DGKI
FOS
SYCP2
POPDC3
EGR1
CCDC33
MGST1
NKIRAS1
COL3A1
VAV3
KIF5A
FHAD1
PCED1A
SH3BP5L
IGFN1
ATF3
COMTD1
PSG3
SYTL5
QPCT
TCN1
RGS20
STC2
TGFBI
FAM171A2
UPP1
IAH1
TGDS
ZNF697
EPSTI1
DENND2A
PTPRB
MX2
CTD-3148I10.9
RBFA
SLCO4A1
NKX2-2
FGF9
ANKMY1
TGFA
BNIP3
WFDC1
ZMYND15
KIAA1211L
PTPRT
RNF31
STC1
NDRG1
RP11-1263C18.3
ASIC2
FRMD4B
ADGRA2
TRIB3
ASNS
SBK1
FAM174A
DALRD3
PLIN2
CR2
DNAJB5
Normoxia1
Normoxia2
Normoxia3
Hypoxia3
Hypoxia1
Hypoxia2

Table S1

gene_symbol	Average Expression	LFC	P.value.adj
hsa-miR-210-3p	2.421453976	2.695186	4.9514E-08
PTPRB	0.2857804	1.738538066	0.005543676
NDRG1	0.532457677	1.702125264	0.007668868
TRIB3	0.384872744	1.55863324	0.034153762
hsa-premir-210	0.157953091	1.473553426	0.047359763
UPP1	1.263767299	1.436378529	0.004207143
ASIC2	1.290199582	1.400179586	0.004207143
STC2	1.682619784	1.319323167	0.001080659
RP11-1275H24.1_nc	1.184162838	1.291471249	0.005273065
BNIP3	1.735724169	1.208848471	0.003381009
RP11-264A11.1_nc	1.640188667	1.195951589	0.004466718
TGFBI	3.248035496	1.130427928	0.000101976
RP11-1263C18.3	3.955616595	1.084339839	0.002144757
TCN1	2.104974419	1.081929627	0.004207143
CAPS	1.459159155	1.045164762	0.047799672
STC1	1.31775539	1.042375239	0.016814205
VEGFA	4.673942346	0.975427284	0.003296336
AMIGO2	1.33992046	0.972580479	0.019954714
CDCP1	2.046699132	0.960792459	0.005273065
DDIT4	1.932661487	0.946294002	0.009436155
LINC00472_nc	1.451304517	0.936879742	0.034674101
SOCS2	1.179392173	0.936728679	0.048708134
RP11-161H23.9_nc	3.114705504	0.924547841	0.004207143
LAMB3	1.715652011	0.898041581	0.010662458
SYTL2	2.054733423	0.877949598	0.019954714
PCDHB3	2.330256867	0.870918063	0.004466718
LOXL2	2.01126249	0.868641257	0.016814205
LOX	2.417221273	0.860667561	0.008495924
P4HA1	3.068209934	0.848657102	0.004207143
KLF7	1.832137892	0.844614805	0.044443859
PSAT1	2.30362157	0.844165441	0.020569585
hsa-premir-3714	2.303416484	0.842444476	0.016814205
ARRDC3	3.425439101	0.816265755	0.001715433
SLC2A3	1.466912986	0.8072138	0.047359763
RND3	5.229575354	0.794879607	0.000164289
XAF1	2.412635359	0.787185719	0.013670586
SCG2	4.347875345	0.776854807	0.017654243
RP11-84G21.1_nc	1.934323191	0.774624836	0.040853285
RP11-356C4.5_nc	3.419297509	0.749209743	0.032469542
CAPG	2.017068533	0.738004192	0.048057067
LDHA	4.99226325	0.736015742	0.004207143
IRS2	2.370240197	0.733404244	0.015246126
MRPS26	2.014542639	0.729088986	0.047799672
PGK1	3.969370246	0.711546794	0.004207143
PFKP	2.218783011	0.701313665	0.027906325
BTG1	2.749320795	0.695206577	0.017654243
SAMD9	2.950375463	0.664606382	0.009333411
AP1S2	3.225755429	0.658683789	0.021573817
ITGB8	4.929417604	0.651178503	0.002144757
MTHFD2	2.980101597	0.624825528	0.034674101
NTM	2.523009971	0.620963675	0.033437791
AUP1	2.471280436	0.59787804	0.047799672
hsa-miR-193b-5p	3.268620594	0.571398148	0.032469542
MEF2A	3.397657219	0.550961031	0.040853285
TPI1	3.792292128	0.515021035	0.034153762
GUCY1A2	3.483361034	0.509933119	0.049285542
TIMP3	5.682755248	0.506362874	0.004466718
ALDOA	3.523764225	0.488242748	0.048435702
MXI1	3.78649179	0.480034086	0.040853285
TXNIP	4.997340224	0.472049994	0.034153762
VIM	8.163455173	0.389934288	0.025474558
IGFBP4	4.275930682	-0.473504232	0.047359763
SFPQ	5.207540845	-0.488228561	0.017552928
MKI67	7.005813655	-0.589263553	0.005543676
EPAS1	4.112701136	-0.63471226	0.013670586
CYP1B1	5.022152007	-0.641963104	0.004207143
